# A Plea for the Kind Reception of the Young Doctor

**Published:** 1914-09

**Authors:** 


### A Plea for the Kind Reception of the Young Doctor.

Oh were you ne'er a schoolboy?
And did you never train?
And feel a swelling of the heart,
You ne'er can feel again?

Have you forgotten when you were given your diploma in medicine how bright and beautiful the world looked and how happy you were and how your heart sang a refrain of good-will to every creature on earth?

It may be you didn't have a cent in the world and were in debt for your education, but you felt that you were fitted for your life-work and you were willing and anxious to apply the knowledge you had gained.

You felt, too, that everyone else would be glad that you had reached the mark to which you had aspired.

Well you found out when you put up your shingle that there were some people who had a grudge against you. And, strange to say, it was the very persons who should have been interested in you because you had chosen the profession that they had embraced. Did this happen to you, doctor, when you first started out to practice medicine? Yes, you fought your way to a good practice, and you can afford to laugh about the matter now, that you are well established in business, but truly down in your heart, would you not feel much more tender toward the old physician who lives in your town, and who has long since ceased to practice, if he had given you a cordial handshake in the beginning, and had said: "My son, the profession you have chosen is a hard one; many thorns and few roses, but, if I can be of any help to you, feel free to call upon me at any time." Now, honest, doctor, wouldn't you have felt more kindly toward him? Of course you would; it's human nature.

Well, sit down and think the matter over and see if you want any brother physician to entertain any but kindest thoughts of you, as life shadows begin to lengthen toward the close.

The Texas State Board of Medical Examiners has just granted a license to practice to 131 young physicians, and it may be some of them will locate in your town.

Won't you be big enough and broad enough to take this young doctor under your wing? Speak a helpful word to him, invite him to the medical society and introduce him to the other physicians. *Yes,* of course, they will look him over just as though he were a new biological specimen, but he will not mind if he feels that you are his friend.

Had it ever occurred to you that many young physicians join the ranks of the quacks because all the regulars frown down on them and try to starve them out?

But thanks be there are some few physicians yet who do lend the helping hand to those just starting whenever the opportunity presents itself.

As an illustration, I wish to mention the fact that Dr. "A," who advertised in the August number of the JOURNAL for a good location, received some half dozen replies from physicians who really wanted to help him to secure a good position. The physician who will take the time to sit down and write a letter to a young doctor who is looking for a location and tell him where his services are wanted is not likely to snub him just because "he thinks he knows it all."

Below is given the list of names of those who successfully passed the examination and were granted a license to practice:

Alverson, D. L., Houston, Texas; Aves, D. R., Seabrook, Texas; Applewhite, H. L., Dallas, Texas; Aiguier, R. L., Birthright, Texas; Brown, S. L., Austin, Texas; Braden, A. H., Beaumont, Texas; Beall, J. R., Decatur, Texas; Bursey, E. H., Weatherford, Texas; Bullard, C. C., Dallas, Texas; Bowyer, O. M., Anson, Texas; Carr, M. M., Dallas, Texas; Crockett, R. H., Houston, Texas; Clark, A. F., Fentress, Texas; Collado, A., Brownsville, Texas; Cooper, J. S., St. Joseph's Infirmary, Houston, Texas; Cook, Clara G., Worcester, Mass.; Collins, E. E., Premont, Texas; Coleman, R. H., Mineola, Texas; Christian, C. H., Smithville, Texas; Clark, C. B., Dallas, Texas; Clements, P. C., Timpson, Texas; Cadenhead, J. F., Fort Worth, Texas; Cupp, C. D., Whitney, Texas; Chandler, S. L., Dallas, Texas; Carlisle, G. L., Dallas, Texas; Denson, J. L., Ben Arnold, Texas; Deason, G. A., Dallas, Texas; Daves, A. C., Hot Springs Ark.; Daughety, J., Dallas, Texas; Dennard, E. A., Mineola, Texas; Davis, B. R., Laredo, Texas; Evans, S. R., Comanche, Texas; Francis, F. W.,

Fort Worth, Texas; Fowler, C. F., Galveston, Texas; Farr, B. H., C., El Paso, Texas; Goode, E. P., Quinlan, Texas; Goodwin, O. P., Dallas, Texas; Guy, W. H., Carbon, Texas; Glass, R. J., Dallas, Texas; Gerardy, H. H., Chandler, Okla.; Grisco, D., Fort Worth, Texas; Gray, C. W., Shiner, Texas; Green, Wm., Terrell, Texas; Gonzales, G. L., El Paso, Texas; Guarjardo, E., Victoria, Texas; Hathhorn, J. D., Bonham, Texas; Hester, W. L., Marshall, Texas; Hewson, J. P., Orange, Texas; Handley, J. J., Dallas, Texas; Hodges, E. D., Waco, Texas; Holder, T. D., Holder, Texas; Hall, J. H., Dallas, Texas; Hammond, J. E., Fort Worth, Texas; Hestard, D. M., Sherman, Texas; Harris, R. A., Dallas, Texas; Jones, J. E., Dublin, Texas; Jackson, H. V., Las Cruces, New Mex.; Jones, S. R., Waco, Texas; Just, F., Burton, Texas; Jenkins, J. G., Temple, Texas; Johnson, R. F., Dallas, Texas; Knight, J. B., Dallas, Texas; Kieke, A. W., Dallas, Texas; Knowles, W. M., Dallas, Texas; King, T. A., Vernon, Texas; Leal, A. J., Brownsville, Texas; Long, G. J., Jr., Del Rio, Texas; Logsdon, W. K., Beeville, Texas; Littlepage, H. B., Fort Worth, Texas; Lipps, P. K., Fort Worth, Texas; Logan, E. J., Silver City, New Mex.; Maxwell, E. S., Nashville, Tenn.; McCreight, W. J., Dallas, Texas; Murphy, C. S., Houston, Texas; Martinez, P., Corpus Christi, Texas; Maffett, M. L., Waco, Texas; Miller, D., Nashville, Tenn.; Martin, J. D., Newton, Texas; McDaniel, R. R., Gorman, Texas; May, J. C., Fort Worth, Texas; McLaurin, J. G., Dallas, Texas; Newton, F. H., St. Louis, Mo.; O'Neil, O. R., Paris, Texas; Puga, A. S., Laredo, Texas; Parke, J. N., Galveston, Texas; Powell, E. V., Galveston, Texas; Puckett, B. M., La Follette, Tenn.; Paloma, V., El Paso, Texas; Padgett, W. O., Dallas, Texas; Padilla, A. G., Brownsville, Texas; Reed, C. R., Sherman, Texas; Roddy, G. H., Alice, Texas; Russell, H., New Franklin, Mo.; Rameriz, J., Laredo; Roan, A. M., Eva, Ala.; Randolph, V., Temple, Texas; Riveroll, C., Laredo, Texas; Randel, B. W., Fort Worth, Texas; Story, F. L., Ennis, Texas; Spiller, S. E., Austin, Texas; Stricklin, C. G., Gustine, Texas; Stansell, I., Dallas, Texas; Scott, K. J., Fort Worth, Texas; Saunders, D. J., Fort Worth, Texas; Singleton, R. O., Wichita, Kan.; Shytles, H. W. G., Venus, Texas; Simms, P. A., Jacksonville, Texas; Swinney, B. A., Newton, Texas; Starnes, W. L., Georgetown, Texas; Smith, W. E., Dallas, Texas; Sparks, J. H., Alto, Texas; Sappington, T. B., Eagle Ford, Texas; Swan, H. A., Abilene, Texas; Thompson, W. T., Texarkana, Texas; Tolson, T. T., Lafayette, La.; Torres, E. L., El Paso, Texas; Webb, J. B.,

Delia, Texas; White, C. M., Diboll, Texas; Woods, D. R., Perrin, Texas; Welch, J. G., Kountze, Texas; Williams, M., Denham Springs, La.; Wynman, G. H., Dallas, Texas; Walter, D. J., Brenham, Texas; Worsham, A. B., Sulphur Springs, Texas; Williams, D. C., Post City, Texas; Whisenant, J. R., San Antonio, Texas; Zuniga, W. O., El Paso, Texas; Zienun, N., Austin, Texas; Vaughan, T. D., Galveston, Texas; Yater, A. D., Cleburne, Texas.

